# Correlations between the histopathological alterations in minor salivary glands and the clinically suspected Sjögren’s syndrome

**DOI:** 10.3389/pore.2023.1610905

**Published:** 2023-05-15

**Authors:** Mária Janka, Attila Zalatnai

**Affiliations:** Department of Pathology and Experimental Cancer Research, Faculty of Medicine, Semmelweis University, Budapest, Hungary

**Keywords:** immunohistochemistry, clinicopathology, retrospective study, Sjögren syndrome, labial gland biopsy

## Abstract

In sicca syndrome patients the xerostomia, xerophthalmia and the serological findings may strongly suggest the autoimmune Sjögren’s syndrome, but the histological findings in the labial salivary gland biopsies do not always justify the suspected diagnosis. The aim of this study was to compare the histomorphological changes and the clinical findings in patients with pathologically established Sjögren’s syndrome and in cases with negative histology. A total of 133 labial biopsies have been retrospectively evaluated from 2015 to May 2022, and the characteristic Sjögren’s lesions were found in 67 cases. According to the clinical data, 34 cases proved to be primary, and 33 were associated (“secondary”) forms. In 66 cases, the histology did not justify Sjögren’s syndrome; a significant acinar loss, fibrolipomatous infiltration, and mild sialadenitis had led to the clinical symptoms. In Sjögren’s histologies, the acinar loss was detected in just 31.8% of cases, which might indicate that the diminished saliva production represents immune-mediated hypofunction rather than direct damage of the acini. This is the first systemic study in Hungary investigating the correlation between pathological alterations and clinical findings.

## Introduction

Sjögren’s syndrome (SS)—named after a Swedish ophthalmologist—is a treatable, but not curable autoimmune disease affecting primarily the salivary and the lacrimal glands, but in about 50% of patients, other immune-mediated diseases are also detected. The primary Sjögren’s syndrome is not associated with other disorders, while the other types are coupled with systemic connective tissue diseases, such as SLE, rheumatoid arthritis, or systemic sclerosis [1]. Although its frequency may vary considerably depending on the classification criteria used, on the clinical or pathological settings, or on the age groups studied, the estimated prevalence of the primary forms in a US population-based cohort was reported as 22/100,000 inhabitants [2], but this figure in Europe is around 39/100,000 [3]. A recent report in Hungary claimed a lower incidence (16/100,000) of newly diagnosed SS cases [4].

Since the autoimmune process harms salivation and tear production, the classical clinical symptoms are characterized by xerostomia and xerophthalmia (sicca syndrome), but sometimes other mucosal dryness can also be experienced (in the digestive tract, vagina, etc.). However, because dry symptoms can also be found in other conditions, such as a side effect of antihypertensive drugs or chemotherapeutic agents, in old age or in diabetes mellitus [5], correct diagnosis of Sjögren’s syndrome requires well-defined criteria. Over the years, several schemes have been proposed both for the primary and the associated forms [6, 7]. In the actual ACR-EULAR classification, the diagnosis is based on the combination of the clinical symptoms, the autoimmune markers, and the histopathological findings, but the SS-A/Ro/positivity and the labial salivary gland biopsy results have higher scores [8].

Although the presenting clinical symptoms indicating Sjögren’s syndrome are usually highly suggestive, salivary gland biopsy is indicated when a patient experiences long-standing (over 3 months) ocular and/or oral dryness with or without accompanied systemic autoimmune diseases to reinforce the diagnosis. The clinical symptoms may poorly correlate with the overt Sjögren’s syndrome [9]. The primary and associated forms may show similar manifestations in SLE, for example, definitive SS is just diagnosed in around 14%–18% [1]. Similarly, in rheumatoid arthritis dry eye, dry mouth, or ocular foreign body sensations were seen in 78%, 68%, or 17% of patients, respectively, however, only 8.7% of patients proved to be Sjögren’s syndrome [10]. Moreover, SS-A/SS-B antibodies can also be detected in a general population (up to 16%) without displaying any autoimmune disease [11].

The major salivary glands are responsible for over 95% of saliva production, while the minor salivary glands have just an ancillary role, but their continuous secretion is important in the moistening of the oral cavity [12]. The histological sampling of the major salivary glands, however, is just exceptionally practiced [13, 14]. The generally accepted method is the labial biopsy. This sensitive technique was introduced by *Chisholm* and *Mason* [15], and they showed a definite association between the clinically suspected SS and the lymphocytic foci in the minor salivary glands. The actual standard diagnostic criteria of the primary Sjögren’s syndrome have been defined by the ACR-EULAR classification [8].

**FIGURE 1:**
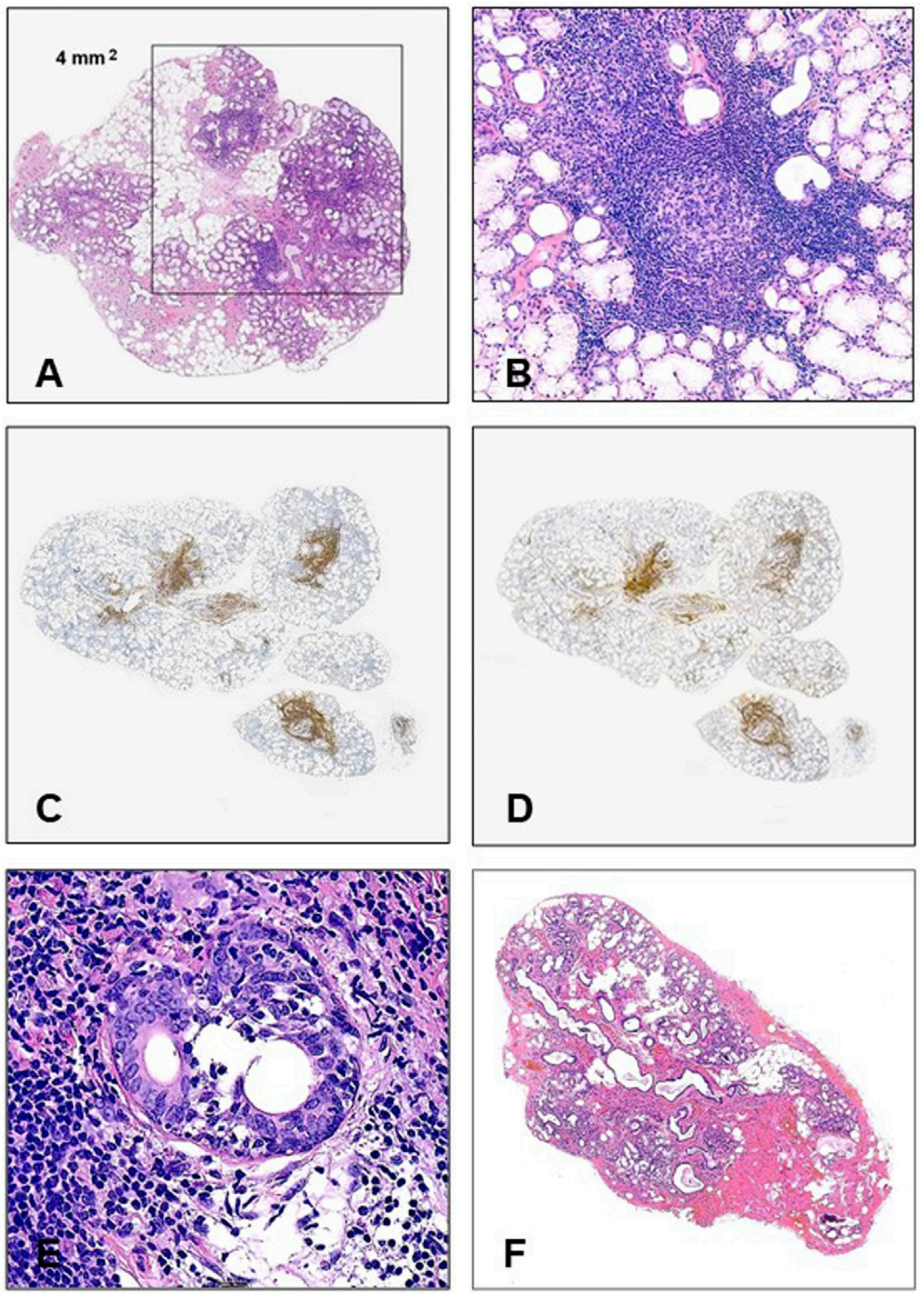
**(A)** For the histological establishment of Sjögren’s syndrome in labial biopsy materials, at least 1 lymphoid focus containing a minimum of 50 mature lymphocytes should be identified in 4 mm^2^ (HE, ×20). **(B)** Germinal center in a minor salivary gland (HE, ×200). **(C)** CD20 positivity in the small labial gland (×20). **(D)** CD3 positivity in small labial gland (×20). **(E)** Small duct is infiltrated by mature lymphocytes (HE, ×400). **(F)** Typical histology in a non-Sjögren’s labial biopsy (HE, x20).

In this classification scheme the anti-SSA/Ro positivity and the labial biopsy results have the highest weight. Histologically, the diffuse lymphocytic infiltration is not a feature, it represents just an aspecific sialadenitis; for establishing Sjögren’s pathology the mature lymphoid cells (at least 50) must be arranged in foci, at least one focus per 4 square mm (Figure 1A), sometimes with secondary follicle formation with germinal center (Figure 1B). Although many innate and adaptive immune cells play a role in the damage of the glandular structures [16], the foci are composed of subsets of B-cells associated with T-cell population [17, 18] (Figures 1C, D). In the glandular damage the interplay between the immune cells and the glandular elements seems to be important [19], but the hypofunction rather than destruction is regarded as the main mechanism of the secretory failure [20].

Although by definition the histological hallmark of Sjögren’s syndrome in labial biopsies is ≥1 focus per 4 square mm, it would be worth investigating other morphological alterations in the glands, moreover, whether the extent of the lymphoid accumulation correlates with the clinical symptoms.

## Materials and methods

Between 2015 and May 2022, 133 labial biopsies have been evaluated at the (1st) Department of Pathology and Experimental Cancer Research. The patients with clinically suspected Sjögren’s syndrome had an average age of 58.5 (25–82) years. The vast majority of patients were female (125/133 = 94%). The clinical data have been collected from the Semmelweis University electronic archives and all patients provided their written informed consent. The patients with previous head/neck irradiation, the HCV-, IgG4-, COVID-19 or HIV-positive cases, the organ-, or bone marrow transplanted patients have been excluded, and similarly, those who regularly took antidepressants or anticholinergic drugs. Non-insulin-dependent, but well-controlled diabetes mellitus was mentioned in 6 cases. Malignant tumors were diagnosed in three patients (breast cancer, malignant melanoma, and MALT-lymphoma), but none of them were on an actual chemotherapeutic regimen. Before taking a labial gland biopsy, a detailed immunological checkup was accomplished and in the oral cavity no preneoplastic or infective conditions were detected. During the histological evaluation the following features were assessed: the number of lobules, the percentage of the infiltrating fat and connective tissue, the rate of the acinar loss, the ductular changes, the small vessels, and the lymphocytic foci. The proportions of the components have been assessed by either routine ×20 microscopic field and calculated to square mm, or the scanned slides were evaluated by using a SlideViewer program (P1000 Scanner with Panoramic 1,000 software; 3D Histech Ltd., Budapest). Both investigators (MJ, AZ) double-checked the histological findings. The establishment of Sjögren’s histology was based on the internationally accepted consensus [21] (Figure 1A), and among the 133 biopsies, 67 specimens (50.4%) fulfilled these criteria. In the remaining 66 cases, the clinical settings indicated SS, but in the labial biopsies, the required criteria were not found. In both groups the focus scores have been calculated for 4 square mm glandular areas.

The aim of this study was to evaluate the morphological changes of labial gland biopsies in patients with clinically suspected sicca syndrome, to compare the findings with Sjögren’s and non-Sjögren’s cases, and to analyze the relevant clinical data.

## Results

### Sjögren’s histologies

The histological changes are presented in Table 1. The labial glands contained 2–16 lobules. The histologically visible glandular damages were highly inconstant, with the only universal finding being the presence of the diagnostic (≥1) lymphoid foci. The average number of foci per gland was 3.6, varying between 1 and 11, and the lymphoid accumulations were arranged around the moderately distended ductules (Figure 1A). In two cases germinal centers were also formed (Figure 1B). The foci have been composed of proportional CD20^+^ and CD3^+^ mature lymphocytes (Figures 1C, D), among the T-cells with the helper (CD4^+^) predominance. In a single, severe case the lymphocytes invaded some ductular cells (Figure 1E), but the attack of the acinar elements was not the feature. Loss of the acini was detected in about one-third of cases and these areas were mainly replaced by fibrosis (89.5% of cases) and partly by fat tissue (67.1%). The extent of the glandular injury was highly variable, ranging from 5 to 80 percent. The remaining glands were structurally preserved, and no apoptotic activity was seen. The accumulation of connective tissue led to dilatation of the small ducts in about half of the cases, accompanied by inspissated secreted material. In four samples, mild vasculitis was also seen close to the lymphoid foci.

**TABLE 1:** Histopathological findings of labial gland biopsies in Sjögren’s and non-Sjögren’s syndrome patients.

	Sjögren’s syndrome	Percent	Non-Sjögren’s syndrome	Percent	P
Focus score ≥1	67/67 average focus number: 3.6 (1–11)	100	0/66	0	
Acinar loss	21/67 (5%–80%)	31.3	50/66 (20%–60%)	75.8	0.025^a^
Fat infiltration	45/67 median area: 32.5% (0–85)	67.2	60/66 median area: 25% (15–60)	91	NS
Fibrosis	60/67 median area: 22.5% (0–50)	89.5	64/66 median area: 50% (40–67)	97	NS
Ductectasia	37/67	55.2	49/66	74.2	NS
Vasculitis	4/67	6	0/66	0	
Aspecific chronic sialadenitis	0/67	0	50/66	75.8	
Normal histology	0/67	0	7/66	10.6	

^a^ Calculated by chi-square test.

### Non-Sjögren’s histologies

There were 66 patients who complained of chronic xerostomia and/or xerophthalmia and were clinically suspected of having Sjögren’s syndrome, but the labial gland biopsies did not show the diagnostic lymphoid foci (Table 1; Figure 1F). Instead, a frequent finding was the mild, aspecific sialadenitis in the stroma, displaying a loose, but diffuse lymphoplasmocytic infiltration, without neutrophils. The inflammatory cells did not attack the acinar elements. The predominating histological finding was the significant accumulation of the fibrolipomatous stroma, the small ducts had been dilated in two-thirds of samples, and more than two-thirds of the acini had been severely atrophied or disappeared. Signs of necrosis or apoptosis were not seen. In seven samples (10.6%), no histological abnormalities were found at all.

### Clinical and laboratory findings in patients with Sjögren’s histologies

**FIGURE 2:**
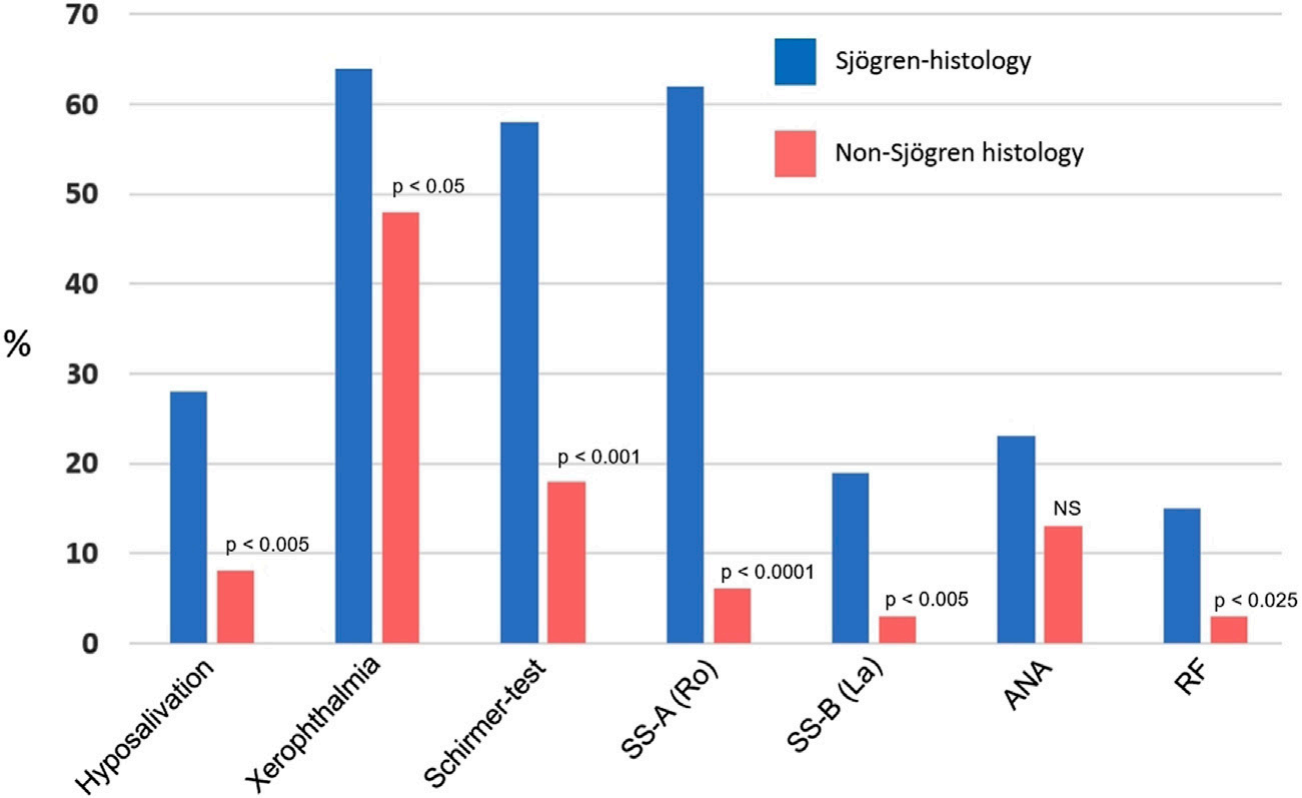
Clinical and laboratory findings in patients with histologically proven Sjögren’s syndrome and in non-Sjögren’s histologies. ANA, antinuclear antibody; RF, rheumatoid factor (Significances were calculated by Chi-Square test).

The relevant clinical data are illustrated in Table 2 and in Figure 2. Although all patients complained of subjective chronic dry mouth sensation, however, objective hyposalivation measured by unstimulated sialometry was only found in less than one-third of cases. Xerophthalmia was also a rather common symptom, which was frequently evidenced by the Schirmer-test (reduced tear production). Interestingly, the ocular symptoms were not always equally affecting both eyes. The presence of the SS-A autoantibody was a frequent, but not a constant finding in all patients (62%), but the SS-B (which is a less sensitive marker), being detectable just in 19%. The non-specific antibodies (ANA, RF) were identified in 23% and 15%, respectively, however, among them no SLE or rheumatoid arthritis were diagnosed.

**TABLE 2:** Clinicopathological findings in patients with or without typical Sjögren-histologies.

	Sjögren (N = 67)		Non-Sjögren (N = 66)		
	No.	%	No.	%	P
Hyposalivation	19/67	28	5/66	8	<0.005
		Sensitivity: 28.3%			
		Specificity: 92%			
Xerophthalmia	43/67	64	32/66	48	<0.05
		Sensitivity: 64.2%			
		Specificity: 51.5%			
Schirmer-pos.	39/67	58	12/66	18	<0.001
		Sensitivity: 58.2%			
		Specificity: 81.8%			
SS-A (Ro52)	42/67	62	4/66	6	<0.0001
		Sensitivity: 62.7%			
		Specificity: 93.9%			
SS-B (La)	13/67	19	2/66	3	<0.005
		Sensitivity: 19.4%			
		Specificity: 97%			
ANA	15/67	23	9/66	13	NS
		Sensitivity: 22.4%			
		Specificity: 86.3%			
RF	10/67	15	2/66	3	<0.025
		Sensitivity: 14.9%			
		Specificity: 97%			

In all the 67 labial gland biopsies the focus score fulfilled the diagnostic criteria, however, it was found that the number of lymphoid foci was rather variable. Therefore, we compared this figure with the clinical data but no correlation was found. The dry symptoms occurred at the same frequency irrespective of the extent of the lymphoid infiltration. The SS-A-positivities did not differ either.

Although prolonged xerostomia is also a frequent finding in poorly controlled diabetes mellitus, among primary Sjögren’s patients only one, well-managed diabetic patient was noted. Other, occasional, probably unrelated diseases were: polyneuropathy (*n* = 1), trigeminal neuropathy (*n* = 1), primary biliary cholangitis (*n* = 1), idiopathic thrombocytopenic purpura (*n* = 1), or antiphospholipid syndrome (*n* = 1). The overwhelming majority of our cases were not associated with systemic autoimmune conditions.

### Clinical and laboratory findings in patients with non-Sjögren’s histologies

Although the presenting signs were also chronic dryness in the mouth, objective hyposalivation was seen in less than 10% of cases (Figure 2). Albeit xerophthalmia was a frequent complaint (48%), it was objectively reinforced by Schirmer-test in just 18% of cases. SS-A and SS-B have rarely been detected (6% and 3%, respectively), and similarly, rheumatoid factor was seldom noticed. All these figures differed significantly from those of the values seen in Sjögren’s histologies. Antinuclear antibody was a relatively frequent finding (13%).

Within these patients, none of them took antidepressants or anticholinergic drugs. Diabetes mellitus was only diagnosed in five cases, but autoimmune diseases were frequently mentioned: 8 rheumatoid arthritis, 3 SLE, 1 DLE, 2 Hashimoto thyroiditis, 11 hypothyroidisms, and 5 non-differentiated autoimmune disease. In addition, 13 patients complained of various rheumatic symptoms, but without evidenced autoimmune background.

## Discussion

No single clinical or laboratory finding is characteristic for Sjögren’s syndrome. The diagnosis is based on a subjective and objective decrease of saliva and tear productions, the presence of specific autoantibodies (SS-A, SS-B), and pathological evaluation of the minor salivary glands. All these findings are scored (summing up 6 points), and the disease can be established, when the scores are ≥4. Although the positive labial biopsy scored 3, histology alone is not a gold standard, but it serves a pivotal role in the diagnostic workup; the most important morphological sign is the presence of ≥1 lymphoid focus per 4 square mm, but other, additional features are also useful suggesting signs. Based on a large scale, prospective cohort study (1726 labial gland biopsies), however, the clinical/lab findings may not always correlate with the morphological alterations [22]. Similar data have also been provided by a Hungarian study [4], where a positive focus score was only found in 109 cases (31.8%) out of 342 biopsies from patients with Sicca symptoms.

In our study, loss of the acini in Sjögren’s patients was noted in about one-third of cases, but its extent proved to be highly variable. Although the accumulation of the connective tissue and the fat cells was a very frequent finding, they seemed to be consecutive and not causative events. The lymphoid foci, containing equally CD20^+^ and CD3^+^ lymphocytes, were situated mostly in periductular localization, no direct contact was observable with the acinar structures, and morphological signs of necrosis/apoptosis were not seen either. Direct invasion of the small duct was just seen in a single, severe case (Figure 1D). The clinical symptoms or the SS-A positivity did not correlate with the severity of the focus score. These findings are consistent with those by *Venables* [20], who claimed that hypofunction rather than the direct glandular destruction can be regarded as the main mechanism in the disease.

In Sjögren’s disease the characteristic lymphoid foci are comprised of many types of immune cells and their interaction with the epithelial elements is an important issue regarding glandular destruction [16, 20, 23]. The proportion of the T-and B lymphocytes, however, is not constant throughout the disease course: in the early stage CD4^+^ cells predominate, and the B-cells appear later, but they mutually interact [19]. The interferon-γ, IL-17, IL-21, or the overstimulated B-cell products (excessive amounts of various antibodies) play multifaceted roles [24], and these findings have initiated therapeutic attempts at B-cell depletion. Although these strategies are logical and encouraging, the results are still indecisive [18, 25, 26]. Moreover, some innovative and promising novel trials are also under investigation [27].

In 66 labial gland biopsies, focal lymphoid collections were never seen, just a diffusely occurring, mild, aspecific sialadenitis, albeit in these patients the clinical symptoms suggested Sjögren’s syndrome. While in Sjögren-histologies acinar loss was a feature in only approximately one-third of cases (21/67), in non-Sjögren’s biopsies the acini did disappear in around 76% of cases (50/66), replaced by fibrolipomatous stromal elements in 91%–97%. Due to the compression effect, the small ducts were dilated, with some secreted material in their lumina. Interestingly, in 7 out of 66 biopsies (10.6%) the labial glands displayed a normal morphology.

It seemed to be perplexing the detection of the SS-A(Ro) antibody in patients whose labial biopsies did not show the histological features of Sjögren’s syndrome (4/66, 6%), raising the likelihood that these cases could have been misdiagnosed. However, this antibody is not an absolute marker, it can often be detected in other autoimmune disorders [28–30]. Moreover, some authors have found that anti-SSA or anti-SSB antibodies can be present at a high percentage in the early phase of Sjögren’s syndrome, even years before the clinically manifested disease, or in seemingly healthy people [27, 31]. In our study, among the cases presenting dry syndromes and SS-A(Ro) positivity but where the labial gland biopsy failed to show ≥1 focus score, there were patients suffering from various rheumatic complaints but definite autoimmune diseases have not been diagnosed. This might explain the unexpected lab finding.

In conclusion, the clinical symptoms suggesting Sjögren’s syndrome always require histological investigation of the small labial salivary glands to evaluate the morphological alterations reinforcing or excluding the supposed diagnosis. It was clear from the clinicopathological findings that neither the objective measurements of the saliva or tear production or the presence of autoantibodies reached high sensitivities alone, although xerophthalmia and the SS-A had a highest suggestive value. For that reason, in the ACR/EULAR consensus the Ro antibody and the histology get the highest weight (3-3 points each).

While in Sjögren’s histologies acinar loss was seen in just one-third of cases, no direct contact was found between the glands and the lymphoid foci, and because no necrosis or apoptosis were the feature of damage, it is presumable that the hyposalivation resulted from the indirect effect of the lymphoid foci. This view is further reinforced by the negative connection between the clinical signs and the number of foci. When the biopsies did not justify the sicca syndrome, definitive reduction of the acinar structures and heavy accumulation of fibrolipomatous tissue led to decreased saliva production.

This research is the first systemic study in Hungary investigating the correlation between pathological alterations and the clinical findings.

## Data Availability

The raw data supporting the conclusion of this article will be made available by the authors, without undue reservation.
